# The impact of the gut microbiome on tumor immunotherapy: from mechanism to application strategies

**DOI:** 10.1186/s13578-023-01135-y

**Published:** 2023-10-13

**Authors:** Ciliang Guo, Lingkai Kong, Lingjun Xiao, Kua Liu, Huawei Cui, Qilei Xin, Xiaosong Gu, Chunping Jiang, Junhua Wu

**Affiliations:** 1 State Key Laboratory of Pharmaceutical Biotechnology, Jiangsu Key Laboratory of Molecular Medicine, Medical School of Nanjing University, National Institute of Healthcare Data Science at Nanjing University, Nanjing University, 22 Hankou Road, Nanjing, 210093 Jiangsu China; 2 Jinan Microecological Biomedicine Shandong Laboratory, Shounuo City Light West Block, Qingdao Road 3716#, Huaiyin District, Jinan, Shandong China

**Keywords:** Tumor immunotherapy, Microbiome, Immunity, Immune checkpoint blockade, Adoptive T-cell therapy, Probiotics, Prebiotics, Fecal microbiota transplantation

## Abstract

Immunotherapy is one of the fastest developing areas in the field of oncology. Many immunological treatment strategies for refractory tumors have been approved and marketed. Nevertheless, much clinical and preclinical experimental evidence has shown that the efficacy of immunotherapy in tumor treatment varies markedly among individuals. The commensal microbiome mainly colonizes the intestinal lumen in humans, is affected by a variety of factors and exhibits individual variation. Moreover, the gut is considered the largest immune organ of the body due to its influence on the immune system. In the last few decades, with the development of next-generation sequencing (NGS) techniques and in-depth research, the view that the gut microbiota intervenes in antitumor immunotherapy through the immune system has been gradually confirmed. Here, we review important studies published in recent years focusing on the influences of microbiota on immune system and the progression of malignancy. Furthermore, we discuss the mechanism by which microbiota affect tumor immunotherapy, including immune checkpoint blockade (ICB) and adoptive T-cell therapy (ACT), and strategies for modulating the microbial composition to facilitate the antitumor immune response. Finally, opportunity and some challenges are mentioned to enable a more systematic understanding of tumor treatment in the future and promote basic research and clinical application in related fields.

## Introduction

Cancer is the second leading cause of human death. The prevention and control of cancer is still challenging. Population expansion and aging, which have been reflected in the growth rate of the number of cancer diagnoses and deaths due to cancer, inevitably challenge the process of rapid social and economic development and are important indicators of its quality. With the increasing incidence of cancer, research on its treatment has long been a popular and difficult topic in modern biology and medicine. Traditional tumor treatment methods, including chemotherapy, radiotherapy and surgical resection, are prone to drug resistance, have a high recurrence rate and greatly harm the patient’s body, which limits their prognostic effect. Therefore, new tumor treatment strategies that induce less resistance are urgently needed. Over the past few decades, a large amount of basic experimental data and clinical trial results have shown that immunotherapy has the potential to change this situation. However, these treatments have different outcomes in different patients; some patients experience lasting benefits, and others do not benefit at all, which limits their clinical application [1–4]. This situation makes the development of innovative combination therapy to overcome drug resistance and improve the response rate an important aspect of tumor therapy research.

With the progress and development of whole genome sequencing technology, a substantial amount of evidence has shown that the microbiota in the human body has an important influence on the effect of tumor therapy [5, 6]. It is estimated that a normal adult can be colonized with up to 3 × 10^13^ commensal microbial cells, totaling over 3000 species, with over 97% colonizing the colon and the rest distributed throughout the body [7, 8]. A variety of factors can influence the symbiotic microbial composition within an individual, such as the composition of the maternal flora, the manner in which the infant is delivered, diet, exposure to antibiotics or other drugs, lifestyle, and environmental factors [9]. Thus, in contrast to the relative uniformity of microbes among different individuals at the phylum level, the composition of gut microbes at the species level varies greatly among individuals, making it difficult for researchers to define the composition of the core healthy gut microbes in humans. This challenge suggests that it may be more appropriate to define indicators of a core healthy microbiome based on the microbial function indicated by the presence of genes involved in microbial metabolic pathways [10, 11].

Commensal microbiomes are closely related to body health, and their disorders can lead to a variety of diseases [12, 13]. Increasing attention has been given to the relationship between the gut microbiome and the occurrence and development of tumors [14–16]. In addition, with the in-depth study of tumor therapy in recent years, a large amount of data has shown that the gut microbiota has a considerable impact on the results of the treatment of various types of tumors, including lung and kidney cancers [17], melanoma [18], and colorectal cancer [19]. Most studies have confirmed that the gut microbiota affects the antitumor response by influencing the immune system. In response to these findings, several strategies have been developed to alter gut microbiome composition and ultimately prolong progression-free survival (PFS) and overall survival (OS) in patients.

Here, we review important studies published in recent years focusing on the influences of the microbiota on the maturation of the immune system. Furthermore, we emphasize the microbiota and the mechanisms underlying its effects on tumor immunotherapy, including ICB and ACT; we also highlight strategies that shape the microbial composition to facilitate the antitumor immune response to enable a more systematic understanding of tumor treatment in the future and promote basic research and clinical application in related fields.

## Gut microbiome and tumor progression

One of the hallmarks of malignant tumors is gene instability and mutation [20, 21], and certain gut microbes that can induce gene mutation have an important influence on the occurrence and progression of tumors, especially in the gastrointestinal system [22, 23]. Regarding the specific mechanism of microbes affecting tumors, current research results mainly support two modes of action: direct and indirect carcinogenic effects. Some bacteria have direct carcinogenic effects and are known as carcinogenic microorganisms. For example, *Helicobacter pylori* can produce viral factors, including urease, which act on epithelial cells in gastric pits, leading to endoplasmic reticulum stress, autophagy, oxidative stress and other inflammatory reactions, thus promoting the pathological changes of gastric tissues, which may develop into gastric cancer [24].

Similarly, *Salmonella Typhi* bacteria that colonize the gallbladder can also produce a cancer-causing typhoid toxin, which causes DNA damage and cell cycle changes in gallbladder cells, leading to gallbladder cancer[25]. Enterotoxigenic *Bacteroides fragilis* (ETBF) has been associated with the induction of colitis and colon tumorigenesis [26]. The toxin produced by ETBF can lead to chronic inflammation in CRC. Mechanistically, ETBF can migrate from the intestinal tract and localize to the mammary gland, where it induces epithelial cell proliferation and promotes tumor growth and metastasis in a Toll-like receptor 4 (TLR4)-dependent pathway [27, 28]. *Fusobacterium nucleatum* adheres to colon tissues through its unique FadA adhesin, which binds to E-cadherin on the surface of colon cells and activates β-catenin signaling and Annexin A1, resulting in inflammatory and carcinogenic responses [29, 30].

In addition, carcinogenic pathogens can also induce tumorigenesis indirectly through immune cells of the tumor microenvironment. *F. nucleatum* can express Fap2 protein in the tumor microenvironment, which binds to the T-cell immunoglobulin and ITIM domain (TIGIT) receptor of immune cells and inhibits the cytotoxicity of natural killer (NK) cells and activation of T cells, thus producing immunosuppressive effects and promoting tumor growth and metastasis [31]. In recent impressive research reports, Pushalkar et al*.* induced immunogenicity reprogramming of the tumor microenvironment by reducing the microbial load in pancreatic tumors, including a reduction in myeloid-derived suppressor cells (MDSCs) and increased differentiation of M1-type macrophages, and promoted CD4^+^ T-cell differentiation and CD8^+^ T-cell activation, thus improving the effect of tumor treatment [32]. Ma and colleagues found that bile acid (BA) produced by intestinal microbial metabolism could act as a messenger to control the accumulation of chemokine-dependent NKT cells in the liver and promote liver-specific antitumor immunity [33]. Short-chain fatty acids (SCFAs) could lead to an increase in the number of Tregs in the colon, as well as the production of the anti-inflammatory cytokines interleukin-10 (IL-10) and transforming growth factor-β (TGF-β), which inhibit the development of tumors [34]. In GF- or antibiotic-treated mice, Kras mutation and p53 gene deletion cannot induce lung cancer because pulmonary symbiotic bacteria can induce the proliferation and activation of γδT cells and promote the development of inflammatory tumors through the local release of IL-17 and IL-23 [35]. Another study also found that p53 mutations induced cancer only in the presence of gallic acid, which was produced by commensal bacteria [36]. All this evidence suggests that intestinal microbes play an important role in tumor progression.

According to the current research results, most of the microbes that promote tumor progression by inducing gene instability are somewhat specific microbial species and their toxic proteins, and the disturbance of intestinal microecology or increased microbial load in the tumor microenvironment is often related to the inhibition of antitumor immunity. However, the fine regulation between intestinal microecological disorders and antitumor immune responses has not been thoroughly elucidated, and most research results lack more general trends and characteristics, which may be due to the limitation of the research scale or differences in research methods.

## Microbiome-based tumor diagnosis

In view of the nonnegligible influence of the microbiome on tumor progression, identifying microbial characteristics is a valid means to diagnose the threat and progression of tumors [37]. CRC and advanced adenoma (AA) are closely related to the gut microbiome, but AA is easier to cancerate. To identify AA from CRC, metagenomic analysis was used to describe the microbiome profile and microbial single nucleotide polymorphism (SNP) characteristics [38]. Another recent study distinguished clinically relevant subtypes of precancerous colorectal polyps, such as tubular adenomas (TAs) and sessile serrated adenomas (SSAs), through microbial signatures from 971 patients [39]. Specifically, TA is associated with a decrease in microbial methanogenesis and mevalonate metabolism, while SSAs exhibit increased NAD, bile acid, and sulfate metabolic potential. This study offers humanized evidence that microbial characteristics can serve as biomarkers of the stage of tumor progression.

In addition to the gut lumen, microbiome-based tumor diagnosis can also be applied broadly. Yang et al. provided a random forest analysis approach based on the oral-gut-tumor microbiome for the early detection of hepatocellular carcinoma (HCC) [40]. The fecal microbiome is also different between cervical cancer patients and healthy controls, with Ruminococcus_2 negatively correlated with cancerous stage [41]. Moreover, with the usage of artificial intelligence, tumor diagnosis has moved to a new phase. Xu and colleagues exploited an artificial intelligence diagnosis model, called DeepMicroCancer, for a broad spectrum of cancer types [42]. Combined with random forest and transfer learning models, DeepMicroCancer covers more than twenty common types of cancer, and the accuracy that could be achieved for blood samples is satisfactory for clinical scenarios.

## Impact of the gut microbiome on the immune system

The commensal microbes distributed throughout the human body maintain a continuous interaction with the host. Many researchers believe that the gut, which exhibits immunoreactions driven by a high density of microbes, is the largest immune organ in mammals [14, 43]. Mammalian immune systems have evolved to fight pathogenic microorganisms due to the interaction between the host and commensal microbes [44]. Early colonization of microbes on mucosal membranes in mammals plays an important role in the development and maturation of the immune system. The individuality and variability of commensal configuration are highest in the first 3 years, during which infants are more susceptible to pathogen infections, and such life-threatening infections rarely occur in adulthood [45].

Germ-free (GF) mice are the most commonly used animal models to study the mechanisms of gut microbes’ influence on immune system development and maturation. The immune system and lymphoid organs are severely impaired in GF mice [46, 47]; these mice exhibit a decreased number of Peyer’s patches, a thinner mucus layer, and a lack of lymphoid follicles in the lamina propria [48]. In addition, widespread defects in monocytes, macrophages and neutrophils have been found in the spleen, bone marrow and liver in GF mice. The numbers of macrophages from both embryonic and bone marrow precursors were found to be reduced in the spleen, suggesting that the gut microbiota has an important influence on the origin and development of bone marrow cells [47, 49]. Immunoregulatory Th17 cells in the lamina propria of the small intestine are absent in GF mice but are inducible upon colonization by segmented filamentous bacteria (SFB) [50]. Early B-cell maturation in the intestinal mucosa is regulated by extracellular signals of symbiotic microorganisms and affects intestinal immunoglobulin repertoires [51]. Hence, the relationship between the microbiota and host is not just characterized by parasitism that drafts nutrients from the host; instead, the symbiotic relationship matures host defenses and immunity.

### The regulatory effect on local immunity

**Fig. 1:**
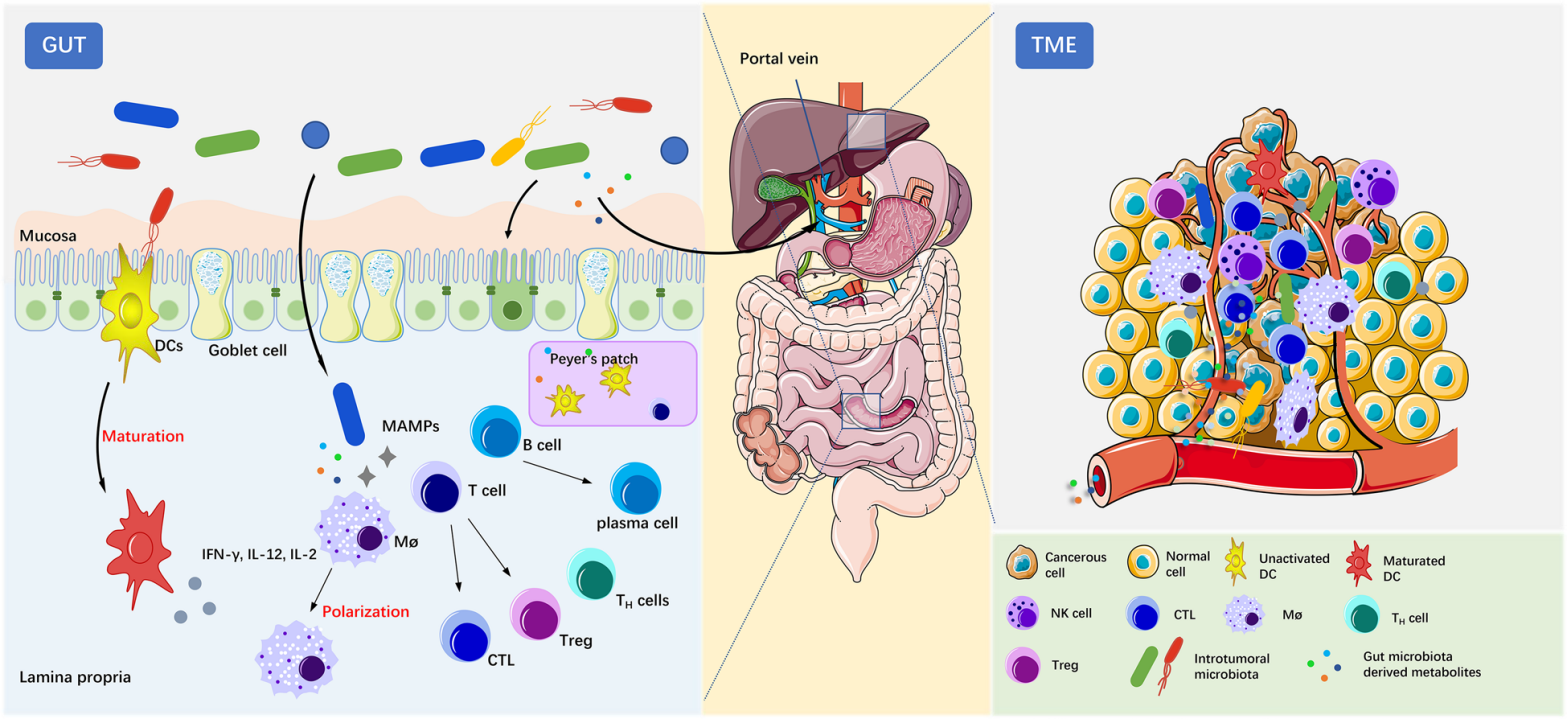
The gut microbiome interacts with the immune system locally in the gut and peripherally in the tumor microenvironment (TME). Within the gut, the gut microbiota plays an essential role in maintaining the mucosal barrier to protect the gut from pathogens. Microbes can interact with DCs directly and induce their maturation. Some microbiota-derived metabolites, such as SCFAs, inosine, and peptidoglycan, or invasive microbes can activate macrophages (Møs), T cells and B cells in the lamina propria or Peyer’s patches. Systemically, gut microbiota-derived metabolites can disseminate to distal sites, especially the TME, through the portal vein and interact with tumor-associated lymphocytes, including DCs, NK cells, Møs and T cells. *CTL* cytotoxic T lymphocyte

A growing number of studies have indicated that the gut microbiota may influence host immunity through multiple mechanisms, including local and systemic immunity (Fig. 1). Locally, the gut microbiota is essential for maintaining the integrity of the mucosal barrier in the intestinal lumen. Disruption of the gut microbiota can lead to a decrease in mucosal barrier function, which results in the entry of pathogenic or normal symbiotic bacteria into the bloodstream and activation of distant pattern recognition receptors (PRRs), triggering an immune response or inflammation [52, 53]. Gut microbes can activate these PRRs, such as Toll-like receptors (TLRs), to signal the immune cells in the gut-associated lymphoid tissue and mesenteric lymph nodes. Microbe-associated molecular patterns (MAMPs) in bacteria, including lipopolysaccharides (LPS) and peptidoglycan, can be presented by macrophages as antigens to Peyer’s patches, where they activate the immune response of antigen-specific B cells and promote the amplification of IgA-secreting plasma cells [54]. In addition, DCs produce tight junction (TJ) proteins between intestinal epithelial cells. At the transepithelial location, DCs can activate TJs and interact directly with bacteria and related molecules in the intestinal lumen to perceive signals [55]. Under conditions of infection, the gut microbiota can activate local phagocytes directly through PPRs to produce cytokines more efficiently [56]. The local interaction between the microbiota and host actuates defenses against most pathogens in the gut lumen and the evolution of immunity, which reemphasizes the existence of symbiotic microbes.

### The regulatory effect on systemic immunity

The gut microbiota can also mediate systemic immune responses by releasing various metabolites into the circulatory system. A key example is SCFAs, which can act on G-protein-coupled receptor (GPCR) signaling pathways or affect epigenetic factors as inhibitors of histone deacetylases (HDACs). SCFAs, such as butyrate and propionate, can induce the differentiation of peripheral Tregs by epigenetic modification of Foxp3 sites [57]. It has also been reported that butyrate is able to increase interferon-γ (IFN-γ) and granzyme B (GZMB) expression in CD8^+^ T cells [58] and induce their transition from an effector phenotype to a memory phenotype [59]. BA is another important immunomodulator produced by microbial metabolism. Studies have shown that BA and its derivatives can control T-cell differentiation and macrophage polarization, especially inhibiting the function of Th17 cells [60], thus regulating the intestinal inflammatory response [61–63]. Generally, the abundance of metabolic genes is irregular among microbes. Thus, these varied metabolites enable the delicate regulation of the immune response by modulating the gut microbiota population.

### The interplay between innate immunity and gut microbiota

Host innate immunity requires not only defense against pathogen invasion but also tolerance to nonpathogenic symbiotic microbiota, that is, maintenance of gut mucosal barrier homeostasis. Various signaling pathways in intestinal epithelial cells and intestinal immune cells play an important role in this process. First, TLRs, which are PRRs, may sense the presence of MAMPs and determine defense or tolerance. For example, polysaccharide A (PSA) from *Bacteroides fragilis* can act on the TIR2/1 heterodimer in cooperation with Dectin-1, thus activating downstream anti-inflammatory immune regulatory genes [64]. NOD-like receptors (NLRs) are also innate immune regulatory sensors. NOD2 inhibits inflammation of the gut by restricting commensal *B. vulgatus* [65].

Apart from PRRs, MyD88 and inflammasomes are also indispensable to host innate immunity for sensing symbiotic microbes and maintaining homeostasis. MyD88 is an adapter for a variety of innate immune receptors that recognize microbial signals. The absence of MyD88 in Treg cells in the small intestine leads to the expansion of Th17 cells, inactivation of the IgA immune response, and initiation of IL-17-dependent intestinal inflammation [66]. The inflammasome induces pyroptosis of infected cells in response to intense pathogen invasion, thus maintaining homeostasis. For example, the NLRP6 inflammasome regulates mucus secretion by “sentinel” goblet cells to prevent pathogenic intruders [67]. In patients with ulcerative colitis, it was found that a symbiotic microbial-induced IgG response and increased activation of FcγR signaling in the colon mucosa jointly induced NLRP3 activation in macrophages and increased production of the proinflammatory cytokine IL-1β [68]. In addition to these immune signaling pathways, innate lymphoid cells (ILCs) are a class of innate immune cells that specialize in rapidly secreting cytokines and chemokines to combat pathogen infection and promote mucosal damage repair [69]. ILCs are characterized by phenotypic diversity and functional plasticity, which are thought to be shaped by different microbial signals. Guo et al. reported that ILC3s can mediate immune surveillance, such as *Citrobacter rodentium*, to facilitate early colonization resistance through ID2-dependent regulation of IL-22 [70].

### The interaction between adaptive immunity and gut microbiota

The interaction between the gut microbiota and adaptive immunity is important in the antitumor T-cell response. Vétizou et al. found that *B. thetaiotaomicron-* or *B. fragilis*-mediated TLR4- and IL-12-dependent Th1 responses were associated with the efficacy of cytotoxic T-lymphocyte-associated antigen 4 (CTLA-4) blockade [71]. Reconstitution of GF mice with commensal microbiota abundant in *Bifidobacterium longum*, *Collinsella aerofaciens*, and *Enterococcus faecium* causes an improved T-cell response and more efficient anti-PD-L1 therapy [72]. *E. hirae* in the gut can translocate into the secondary lymphatic organs, activating the Th17 cell response and promoting the activation of IFN-γ-producing γδT cells, thus improving the therapeutic effect of cyclophosphamide on patients with advanced lung cancer and ovarian cancer [73]. In the study of adaptive immunity induced by gut microbes, DC cells in enteric-associated lymphoid tissue or tumor-draining lymph nodes play an important role in sensing bacteria, presenting bacterial antigens, and secreting cytokines [74].

Symbiotic bacteria activate antitumor T-cell responses by molecular mimicry of tumor-associated antigens, which induces tumor antigen-specific T-cell cross-reactivity. Fluckiger et al. identified a tape measure protein (TMP)-specific H-2K^b^-restricted CD8^+^ T lymphocyte response against a prophage found in the genome of *E. hirae*. In melanoma patients, tumor antigens that are cross-reactive with microbial peptides are recognized by T-cell clones [75]. Similarly, Bessell and colleagues found that T cells targeting epitope SVYRYYGL (SVY), expressed in *B. breve*, cross-react with a model neoantigen, SIYRYYGL (SIY), in B16-SIY. SVY-specific T cells recognized SIY-expressing melanomas in vivo and led to beneficial outcomes [76]. Moreover, human leukocyte antigen (HLA) molecules of both glioblastoma tissues and tumor cell lines present bacteria-specific peptides, which are recognized by tumor-infiltrating lymphocyte (TIL) CD4^+^ T-cell clones [77]. During homeostasis, *Akkermansia muciniphila* (AKK) can induce T follicular helper cells in Peyer’s patches to produce an IgG1 antibody-dependent immune response [78]. Moreover, metabolites produced by bacteria, typically SCFAs [74] or tryptophan derivatives [79], can also induce T-cell- or DC cell-dependent adaptive immune responses.

Therefore, the presence of microbes in the gut allows the immune system to balance the toleration of beneficial microbes and the defense against pathogens. The maintenance of this equilibrium is also influenced by the health state of the host and the stability of the microecology. Due to the close relationship between the gut microbiota and the immune system, there is clearly an important correlation between these commensal organisms and the efficiency of immune-related therapy. Several studies have found that gut microbiome disturbances could affect local and systemic antitumor immune responses [80–82]. In proportion, changing the immune response state by regulating the composition of intestinal microbes may be an effective strategy to improve the efficiency of the treatment of different tumors.

## The gut microbiome in tumor immunotherapy

The rapid proliferation of tumor cells is partly believed to be caused by the failure of immune control. Tumor cells evade the surveillance of the host immune system through various mechanisms, such as downregulating target antigens or creating a TME with immunosuppressive characteristics [83, 84]. Tumor immunotherapy enhances or rebuilds the immune system to monitor, recognize and destroy tumor cells, thereby eventually prolonging the survival of patients [85, 86]. Immunotherapy has significantly prolonged survival and improved quality of life for many cancer patients in whom chemotherapy or radiotherapy regimens have failed [87, 88]. However, with the increase in the clinical application of immunotherapy, researchers have gradually found that the therapeutic effect of immunotherapy on tumor patients varies among individuals. Stool samples from clinical patients who were subjected to sequencing analysis revealed that the features of the gut microbiome and treatment effect exhibited a significant correlation (Table 1), implying that the gut microbiome has a significant impact on tumor therapy. There may be an internal mechanism that is involved in the relationship between the individual diversity of the gut microbiota and the heterogeneity of antitumor immunotherapy outcomes. However, current mechanistic research is mainly focused on the preclinical phase, and the implementation of research findings into clinical application has remained very limited.

**Table 1:** Clinical study of gut microbiome effects on immunotherapy

Tumor type	Number of included patients	Immunotherapy treatment	Favorable clinical outcomes	Unfavorable clinical outcomes	References
NSCLC	65	ICB: αPD-1 mAb; αPD-L1 mAb; αCTLA-4 mAb	*Ruminococcus*; *Akkermansia*; *Faecalibacterium*		[89]
Hepatocellular carcinoma (HCC)	11	ICB: αCTLA-4 mAb; αPD-L1 mAb	*Akkermansia*	*Enterobacteriaceae*	[90]
Lung cancer	46	ICB: αPD-1 mAb		*Fusobacterium*	[91]
Advanced hepatobiliary cancers	65	ICB: αPD-1 mAb	*Lachnospiraceae bacterium*-GAM79; *Alistipes* sp. *Marseille*-P5997; *Ruminococcus calidus*; *Erysipelotichaceae bacterium*-GAM147	*Veillonellaceae*	[92]
NSCLC	30	ICB: αPD-1 mAb; αPD-L1 mAb		*Gammaproteobacteria*	[93]
CRC	39	Chemotherapy alone (oxaliplatin or capecitabine), or combined with ACT	*Bifidobacterium*; *Lactobacillus*; *Enterococcus*	*Dialister pneumosintes*; *Escherichia coli*; *Lactobacillus reteri*; *Streptococcus mutans*; *Enterococcus faecium*; *Streptococcus gordonii*; *Veillonella atypica*; *Granulicatella* sp.; *Trchuris trichiura*	[94]
Advanced HCC	8	ICB: αPD-1 mAb (nivolumab)	*Citrobacter freundii*; *Azospirillum* sp.; *Enterococcus durans*; *Akkermansia*;		[95]
Melanoma	77	ICB: αCTLA-4 mAb and αPD-1 mAb combined		*Bacteroides intestinalis*	[96]
NSCLC	88	ICB: αPD-1 mAb		*Helicobacter pylori*	[97]
NSCLC	75	ICB: αPD-1 mAb	*Streptococcus*		[98]
NSCLC	69	ICB: αPD-1 mAb; αPD-L1 mAb	*Phascolarctobacterium*	*Dialister*	[99]
Lung cancer	34	ICBs	*Clostridiales*	*Rikenellaceae*	[100]
B-cell non-Hodgkin lymphoma	51	Immunochemotherapy	*Dorea formicigenerans*; *Faecalibacterium prausnitzii*		[101]
Metastatic melanoma	130	ICB: αPD-1 mAb; αPD-L1 mAb; αCTLA-4 mAb; ICB combination	*Faecalibacterium*; *Ruminococcaceae*; *Barnesiella intestinihominis*		[102]
NSCLC	11	ICB: αPD-1 mAb	*Akkermansia muciniphila*; *Rikenellaceae*; *Bacteroides*; *Peptostreptococcaceae*; *Mogibacteriaceae*; *Clostridiaceae*		[103]
Metastatic renal cell carcinoma (mRCC)	21	ICB: αPD-1 mAb	*Akkermansia muciniphila*		[104]
NSCLC	17	ICB: αPD-1 mAb	*Lactobacillus*; *Clostridium*; *Syntrophococcus*	*Bilophila*; *Sutterella*; *Parabacteroides*	[105]
NSCLC	37	ICB: αPD-1 mAb	*Alistipes putredinis*; *Bifidobacterium longum*; *Prevotella copri*		[106]
HCC	8	ICB: αPD-1 mAb	*Akkermansia muciniphila*; *Ruminococcaceae* spp.		[107]
Melanoma	27	ICB: αPD-1 mAb; αPD-L1 mAb; αCTLA-4 mAb; ICB combination	*Faecalibacterium prausnitzii*; *Coprococcus eutactus*; *Prevotella stercorea*; *Streptococcus sanguinis*; *Streptococcus anginosus*; *Lachnospiraceae bacterium*	*Bacteroides ovatus*; *Bacteroides dorei*; *Bacteroides massiliensis*; *Ruminococcus gnavus*; *Blautia producta*	[108]
Melanoma	112	ICB: αPD-1 mAb	*Ruminococcaceae* family		[18]
Metastatic melanoma	42	ICB: αPD-1 mAb	*Bifidobacterium longum*; *Collinsella aerofaciens*; *Enterococcus faecium*		[72]
Gastrointestinal cancer	74	ICB: αPD-1 mAb; αPD-L1 mAb	*Prevotella*; *Ruminococcaceae*; *Lachnospiraceae*		[109]
Thoracic neoplasms	42	ICB: αPD-1 mAb	*Akkermansiaceae*; *Enterococcaceae*; *Enterobacteriaceae*; *Carnobacteriaceae*; *Clostridiales* Family XI bacterial families		[110]
Melanoma	94	ICB: αPD-1 mAb	*Actinobacteria* phylum; *Lachnospiraceae*; *Ruminococcaceae*; *Lachnospiraceae* spp.; *Streptococcaceae* spp.		[111]
NSCLC	294	ICB: αPD-1 mAb	*Bifidobacterium*; *Clostridium butyricum*; lac*t*ic *acid bacteria*		[112]
NSCLC	21	ICB combined with chemotherapy	*Bifidobacterium*; *Escherichia*; *Sarterella*		[113]
HCC	35	ICB: αPD-1 mAb	*Ruminococcus*		[114]
Metastatic castrate resistant prostate cancer (mCRPC)	23	ICB combined with enzalutamide	*Streptococcus salivarius*	*Akkermansia muciniphila*; *Collinsella aerofaciens*	[115]
Melanoma	218	ICB: αCTLA-4 mAb and αPD-1 mAb combined	*Ruminococcaceae*	*Bacteroidaceae*	[116]
Advanced gastric cancer	77	ICB: αPD-1 mAb		*Helicobacter pylori*	[117]
multiple myeloma (MM); acute lympholastic leukemia (ALL); non-Hodgkin lymphoma (NHL)	78	CAR-T	*Faecalibacterium*; *Roseburia*; *Ruminococcus*		[118]
Nasopharyngeal carcinoma (NPC)	57	ICB: αPD-1 mAb		*Blautia wexlera*; *Blautia obeum*; *Erysipelatoclostridium*; *Ruminococcaceae bacterium*; *Ruminococcus* sp. AF46-10NS	[119]
HCC	41	ICB: αPD-1 mAb	*Lachnoclostridium*; *Lachnospiraceae*; *Veillonella*	*Prevotella 9*	[120]

### Immune checkpoint blockade (ICB)

Tumors, as collections of cancerous cells, can be recognized and eliminated by the immune system. However, tumor cells secrete inhibitors that recognize and bind to adaptive immune cell surface receptors and inhibit their immune response to tumor cells [121]. ICBs that have been approved by the U.S. Food and Drug Administration (FDA) target two classes of T-cell receptors (TCRs), including CTLA-4 and programmed cell death protein 1 (PD-1) and its ligand (PD-L1). Inhibitory antibodies targeting CTLA-4 [122], PD-1 [123], or PD-L1 [124] can induce antitumor effects in vivo. In addition, many antibodies and small molecule drugs targeting other immunomodulators, including LAG3, TIGIT, TIM3, CD39, CD47 and CD73, are in the process of clinical investigation.

Although ICB therapy improves outcomes for patients with many cancer types, only a portion of patients experience a stable benefit. Even among melanoma patients with the highest response rate to ICB, more than 60–70% of patients do not respond positively to anti-PD-1 antibody therapy; 20–30% of these patients eventually show tumor recurrence and progression [125, 126]. Therefore, there is an urgent need to identify new immunotherapy strategies to improve the immunotherapy response.

There is substantial clinical evidence that the baseline composition of a patient’s gut microbiome is associated with the antitumor efficacy of ICB therapy [17, 81] (Table 1). Notably, the microbes that are favorable and unfavorable to the treatment outcome in different study populations have varied greatly overall, but they may share some important metabolic pathways that allow them to be distinguished. Based on these clinical studies, stool samples from patients with different treatment outcomes were transferred to GF or antibiotic-treated tumor model mice, and the differences in ICB treatment outcomes were paralleled in these mice [18, 72, 127]. Sequencing analysis of fecal samples has often been used to reveal the signature of the bacteria in responding patients, and the signature of beneficial bacteria identified through culture isolation or directly through the use of commercial strain supplements can further confirm the role of key gut microbes in promoting ICB treatment [18, 128]. More strikingly, intestinal bacteria and fungi have opposite effects on tumor therapy, with commensal bacteria essential for an effective antitumor immune response but symbiotic fungi modulating the immunosuppressive microenvironment after treatment. The opposite effect is likely due mainly to fungi, since the size of fungal populations is significantly increased in the gut after bacterial deletion [129]. Although the mechanism by which the microbiome affects tumor therapy is not completely understood, fecal microbiota transplantation (FMT) can be regarded as a therapeutic strategy. However, the future research objective is still to use more specific and direct methods to regulate the microbiota based on a clear understanding of the mechanism of action.

**Fig. 2:**
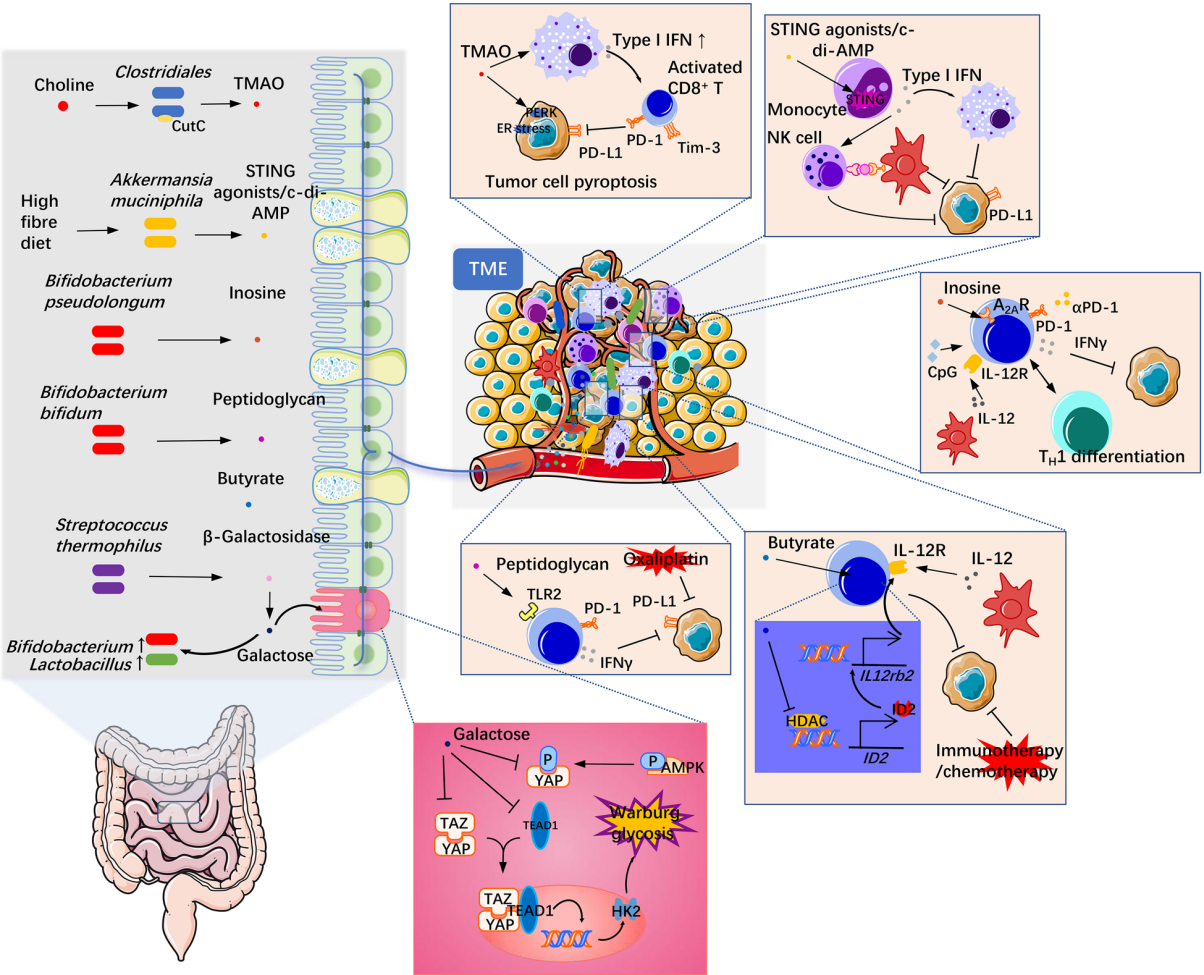
Mechanism through which some gut microbiota-derived metabolites influence antitumor therapy. These metabolites, including TMAO from *Clostridiales*, c-di-AMP from *Akkermansia*, inosine from *Bifidobacterium pseudolongum*, peptidoglycan from *Bifidobacterium bifidum*, β-galactosidase from *Streptococcus thermophilus* and butyrate, are representative metabolites that have been reported in the last two years. They can spread to the TME through the circulatory system or interact with mutated enterocytes directly and mediate antitumor therapy by different mechanisms and pathways. *TLRs* Toll-like receptors, *AhR* aryl hydrocarbon receptor, *A*_*2A*_*R*: adenosine 2A receptor

To date, different studies have found that the key bacterial signatures involved in the ICB response are different, and the mechanisms of action vary as well (Fig. 2). Based on how bacteria exhibit a synergistic antitumor effect with the treatment, the mode of action can be divided as follows: (a) exopolysaccharides [71, 130] or surface proteins [131] in the structure of bacteria themselves can be used as pathogen-associated molecular patterns (PAMPs) to directly stimulate intestinal immune cells and induce innate or adaptive immune responses; (b) metabolites produced by bacteria, such as SCFAs [109, 132, 133], inosine [134], peptidoglycan [127], trimethylamine *N*-oxide (TMAO) [135, 136], neurotransmitters (including dopamine, norepinephrine, serotonin, or γ-aminobutyric acid) [137], ferrichrome [138], β-galactosidase [139], etc., enter the circulatory system through the portal vein to stimulate the TME, changing the immune state and reversing tumor immune tolerance and thus promoting the therapeutic effect of ICBs (Table 2). On this basis, the gut metabolomic profile was characterized in 11 non-small cell lung cancer (NSCLC) patients treated with nivolumab anti-PD-1 therapy, which showed that 2-pentanone and tridecane were significantly associated with early progression, while SCFAs, lysine and nicotinic acid were significantly associated with long-term beneficial effects [140]. Moreover, other studies have found that regulating gut microbial composition and supplementing beneficial bacteria could alleviate immune-related adverse reactions (irAEs) induced by monoclonal antibody therapy targeting CTLA-4 or PD-1 [141, 142], which reiterated the importance of the relationship between the gut microbiome and immunotherapy. Based on these findings, strategies that regulate bacterial populations in the host are reasonable and applicable.

**Table 2:** Reports on microbial metabolites that affect immunotherapy

Tumor type	Immunotherapy treatment	Microbial metabolites	Metabolic bacterial taxa	Target	Overview of mechanism	References
Triple-negative breast cancer (TNBC)	αPD-1 mAb	Trimethylamine N-oxide (TMAO)	*Clostridiales*	PERK	TMAO induced tumor cell pyroptosis by activating ER stress kinase PERK, thus enhancing antitumor immunity mediated by CD8^+^ T cells	[135]
PDAC	αPD-1 mAb and/or αTim-3 mAb	Trimethylamine N-oxide (TMAO)			TMAO induced an immunostimulatory phenotype in macrophages, which supported effector T-cell responses in a type I IFN-dependent manner and facilitated the efficacy of αPD-1 or αTim-3 mAb	[136]
ICI-induced colitis	αCTLA-4 mAb	Indole-3-carboxaldehyde (3-IAld)		AhR	3-IAld activates AhR/IL-22 dependent signaling pathways in the host, controls colon inflammation induced by αCTLA-4 mAb therapy, and reduces adverse reactions associated with ICB therapy by altering the composition and function of the gut microbiota	[143]
Lymphoma (EL4); CRC (MC38); breast cancer (TUBO); melanoma (BRAF^V600E^/PTEN^−/−^)	αPD-1 mAb; αPD-L1 mAb	STING agonists, including c-di-AMP	*Akkermansia muciniphila*	STING signaling pathway	STING agonists generated by bacterial metabolism such as c-di-AMP induce intratumoral monocytes to produce type I IFN, thus promoting the polarization of tumor suppressor macrophages and antigen presentation between NK cells and DC cells, which promotes antitumor immunity	[144]
HNSCC	αPD-L1 mAb	Bacterial lipopeptide Pam3CSK4	*Staphylococcus aureus*	TLR2	Bacterial lipopeptide Pam3CSK4 enhances the expression of PD-L1 in multiple HNSCC cell lines and directly promotes immunosuppression	[145]
Hepatocellular carcinoma (HepG-2)	γδT immunotherapy combined with antibiotics	3-Indolepropionic acid (IPA)			IPA can stimulate γδT cells to release more cytotoxic cytokines, such as granzyme B and perforin, thus improving the efficacy of immunotherapy	[146]
CRC (AOM-DSS/MC38); bladder cancer (MB49); melanoma (B16-F10)	αCTLA-4 mAb	Inosine	*Bifidobacterium pseudolongum*; *Lactobacillus johnsonii*; *Olsenella* species	A_2A_ receptor	Inosine produced by the gut microbiota can translocate to the tumor microenvironment and activated T cells by adenosine A_2A_ receptor combined with costimulation of CpG and IL-12 released by DCs for Th1 differentiation, which resulted in IFN-γ production and enhanced ICB therapy	[134]
CRC (MC38); Lewis lung cancer (LLC1); breast cancer (4T1)	Oxaliplatin; αPD-1 mAb	Peptidoglycan	*Bifidobacterium bifidum*	TLR2	Highly levels of peptidoglycan expressed by *Bifidobacterim bifidum* can act on the TLR2 receptor to stimulate IFN-γ secretion and improve antitumor therapy by αPD-1 mAb or oxaliplatin	[127]
CRC (AOM-DSS)	αCTLA-4 mAb	Lysates	*Lactobacillus acidophilus*		Lysates of *L. acidophilus* decreased Treg and M2 cell levels and increased the proportion of memory CD8^+^ T cells in tumor-draining lymph nodes and mesenteric lymph nodes, thus improving αCTLA-4 mAb efficacy	[147]
Sarcoma (MCA-205); RET melanoma; CRC (CT26/MC38)	αCTLA-4 mAb	Capsular polysaccharide	*Bacteroides thetaiotaomicron*; *Bacteroides fragilis*		The capsular polysaccharides of *Bacteroides thetaiotaomicron* and *Bacteroides fragilis* induce the maturation of lamina propria DCs, which combined with the Th1 immune response induced by IL-12 secretion and promoted the antitumor effect of αCTLA-4 mAb	[71]
CRC (CT26)	αPD-1 mAb	Glycerophospholipid	*Prevotell*; *Akkermansia*		Changes in gut microbiota composition lead to changes in glycerophospholipid metabolism, which affect IFN-γ and IL-2 expression in the tumor microenvironment, resulting in different therapeutic effects of αPD-1 mAb	[148]
CRC (MC38); lymphoma (EG7)	Oxaliplatin	Butyrate		ID2	Butyrate could improve the expression of ID2 by inhibiting HDACs, which promoted IL12R production, and boosted the efficacy of antitumor therapy after stimulation with IL-12 from DCs	[133]

*TAM* tumor associated macrophage, *IL-2/12/22* interleukin-2/12/22

Given the diversity of results, increasing attention is being paid to integrating data from multiple cohorts or to further expanding the cohorts so that the optimal combination of key microbes that further improve the clinical application prospects of strategies targeting the gut microbiota for tumor immunotherapy can be identified.

### Adoptive T-cell therapy (ACT)

The therapeutic effect of ICB is dependent on the presence of preexisting tumor-specific immune cells [149], so its clearance effect is limited in certain tumors with low immunogenicity. Due to this phenomenon, artificial supplementation of tumor-specific immune cells may have a better therapeutic effect. ACT uses autoimmune T cells such as TILs or cytotoxic T lymphocytes (CTLs) to combat cancer. In 1985, transfusions of autologous mature lymphocytes were first reported to produce effective cancer regression [150]. The ACT treatment process typically consists of three steps: (1) isolating and extracting T cells from patient tumor tissues or peripheral blood vessels; (2) culturing and enriching for lymphocytes in vitro; and (3) reinjecting the amplified specific T cells into the patient [151, 152]. With the development of basic biology and immunology, research on the characteristics of immune cells has become increasingly comprehensive. Many studies have shown that T cells produce more specific TCRs or chimeric antigen receptors (CARs) in vitro through gene modification, which could produce a stronger antitumor immune response after infusion into patients [153–155]. Unlike conventional TCRs, CARs recognize antigens independent of major histocompatibility complex (MHC) antigen presentation, avoiding the restriction of MHC molecules and solving the problem of tumor immune escape caused by the inhibition of MHC molecule expression [156, 157]. Recently, ACT, especially CAR-T-cell therapy, has shown excellent efficacy in patients with hematological malignancies and metastatic melanoma [158, 159]. This rapid development of cell therapy brings more hope and possibilities for a cure to patients, but the rate of response to cell therapy also needs to be further improved.

Given the widely accepted role of the gut microbiome in ICB therapy, its impact on CAR-T-cell efficacy was supported by indirect evidence [19, 160, 161] and confirmed in a retrospective cohort study [162]. The study (n = 228) found that antibiotic use during the first 4 weeks of treatment was associated with poorer survival and increased neurotoxicity in B-cell lymphoma and leukemia patients receiving CD19-targeted CAR-T therapy. 16S rRNA and metagenomic sequencing of feces showed that *Ruminococcus*, *Bacteroides* and *Faecalibacterium* were related to the efficacy of CD19 CAR-T treatment. Bacterial metabolic pathways, such as peptidoglycan synthesis and pentose phosphate metabolism, may be biomarkers of CD19 CAR-T-cell efficacy. Moreover, a clinical trial revealed that the irAEs produced in response to CAR-T, especially cytokine release syndrome, are also modulated by the gut microbiome in patients with hematologic malignancies, and similarly, *Faecalibacterium* and *Ruminococcus* are enriched in patients who achieved complete remission [118]. The dominant bacteria and detailed mechanism need further confirmation and exploration. Future application of this mechanism would uniquely enable the stimulation or activation of therapeutic T cells by specific metabolites produced by microbes in vitro before their transplantation into patients.

The relationship between the gut microbiome and ACT response in solid tumors has not yet been reported. However, gut microbes can stimulate tumor cells to secrete a variety of chemokines in patients with colorectal cancer, thus recruiting more T cells into the TME and improving the prognosis of patients. These results revealed the role of the gut commensal microbiota in controlling the extent of tumor invasion by immune cells [19]. In another analysis of plasma samples from colorectal cancer patients treated with ACT in combination with chemotherapy, the abundance of *Bifidobacterium*, *Lactobacillus* and *Enterococcus* in the blood of responders was higher than that in nonresponders [94]. Therefore, using the blood microbiome to predict the effect of tumor immunotherapy may be a more convenient and effective method. In animal models, there were significant differences in the effectiveness of ACT against tumors in mice with similar genes but different origins. Fecal microbiota sequencing showed significant differences in the fecal microbiota composition of mice with different origins, and vancomycin exposure could enhance the therapeutic effect of ACT. This effect was demonstrated by increasing systemic CD8α^+^ DC and IL-12 levels and was associated with more efficient expansion of adoptive antitumor T cells in mice [160]. This study confirmed that it is possible to improve the therapeutic effect of ACT by modulating the gut microbiome composition and promoting the tumor immune response.

Currently, studies on the impact of gut microbes on CAR-T therapy are still in the stage of discovery and validation, but due to the considerable role of the gut microbiome in antitumor immunity, it is believed that there will be increasing evidence to support the effects of this relationship and the specific mechanisms involved.

### Association between the gut microbiome and immunotherapy-related toxicity

Although immunotherapy has achieved remarkable results in the clinical application of tumor patients, there are still a considerable number of cases with toxic reactions, especially irAEs.

In the ICB therapy setting, irAEs correlate with the type of ICB used, such as anti-CTLA4 therapy, which tends to induce colitis and pituitary inflammation, and anti-PD-1 therapy, which tends to induce thyroid dysfunction and pneumonia. The patient baseline gut microbiome has been shown to correlate with the risk of irAEs. In a study of 77 patients with advanced melanoma treated with a combination of CTLA-4 and PD-1 blocking, analysis of blood, tumor, and gut microbes showed that irAEs were not associated with the α diversity of the microbiome but were associated with the baseline abundance of specific bacterial taxa, including *Bacteroides intestinalis* and *Intestinibacter bartlettii*. The abundance of *Bacteroides* was positively correlated with irAEs occurrence and IL-1β levels in the intestinal mucosa, and this conclusion was verified in mouse models [96]. In another study of patients with metastatic melanoma treated with a CTLA-4 and PD-1 blocking combination, *Bacteroides dorei* and *B. vulgatus* were found to be associated with irAEs in a reversed pattern [163]. In a study of 26 patients with metastatic melanoma treated with anti-CTLA-4 monotherapy, the author reported that the baseline abundance of *Faecalibacterium* and other Firmicutes were associated with both treatment efficiency and irAEs, and Bacteroidetes were associated with lower treatment response and lower irAEs [164].

The differences between the two studies may be due to differences in treatment methods or may be the result of functional redundancy between different microbes. In addition, there may be geography-related differences in the microbial communities associated with ICB efficacy and irAEs. Simpson et al. compared fecal microbes of 103 patients with metastatic melanoma from Australia and the Netherlands treated with neoadjuvant ICBs and found that the *Ruminococcaceae* family taxa and AKK were associated with lower treatment effectiveness and more severe irAEs.

In addition, C-reactive protein in peripheral blood can be used as a biomarker for severe irAEs [116]. In the context of immune agonist antibody (IAA) therapy, such as anti-CD40 and anti-CD137, the presence of gut microbiota is correlated with therapeutic toxic reactions such as cytokine release syndrome (CRS), liver damage and colitis. The incidence of toxic reactions was significantly reduced in GF mice or mice treated with antibiotics, while there was no significant effect on the therapeutic efficiency [165]. Although this study did not investigate the function of specific bacterial taxa in depth, the comparison of IAA with ICBs with different effects has implications for intestinal microbial immunotherapy of tumors.

CAR-mediated toxicities are still a problem in CAR-T therapy, including CRS or immune effector cell-associated neurotoxicity. Smith and colleagues reported that antibiotic use was associated with increased neurotoxicity in B-cell lymphoma and leukemia patients treated with CD19-targeted CAR-T therapy [162]. These results not only confirmed the relationship between the intestinal microbiota and the effect and side effects of tumor immunotherapy but also provided a strategic basis for improving the effect of immunotherapy by regulating the intestinal microbiota in different ways.

## Modulation of the gut microbiome to facilitate tumor immunotherapy

The diversity and composition of the gut microbiota are associated with the efficacy of various cancer treatment strategies. In addition to demonstrating the correlation between the two, most studies in this area have demonstrated that the gut microbiota is a therapeutic target, and its modulation is a tool to improve the prospects of clinical application [166]. Large-scale population studies have shown that the diversity of commensal microbial communities in different populations or individuals is largely shaped by environmental factors [167]. With a focus on achieving high clinical compliance or enabling function in daily life, several strategies have been studied to regulate the composition of the gut microbiome to promote immune-related antitumor therapy. The next section reviews the literature related to these studies published in recent years.

### Fecal microbiota transplantation (FMT)

Despite the controversy over its mechanism and safety, FMT has been widely used in the treatment of recurrent *Clostridium difficile* infection (CDI) in the past decade and has achieved good results [168], which also laid the foundation for the therapeutic application of FMT in the treatment of other diseases. Clinical studies on antitumor immunotherapy using the FMT strategy are in the initial stage, and many results have been achieved mainly in animal models [169].

FMT is performed on the premise that the degree of host response to immunotherapy can be transmitted between different individuals through fecal components. Numerous studies have found that GF mice transplanted with stool from patients who had a clinically significant response to ICB treatment are more likely to develop an antitumor immune response to ICB treatment than control mice transplanted with stool from nonresponders. Specifically, tumor progression is slowed, and overall survival is significantly prolonged [18, 72, 170]. These results suggest that the therapeutic outcome of ICB may be influenced by regulating the gut microbiota of tumor patients. More recently, Baruch et al. [128] and Davar et al. [171] verified the efficacy of FMT in anti-PD-1 immunotherapy in patients with metastatic melanoma for the first time in a clinical trial. Two separate studies each observed evidence of clinical benefit in some patients who received FMT treatment. Based on the benefits of FMT in anti-PD-1 therapy in a mouse model [72], Baruch et al. designed a phase I clinical trial (NCT03353402) in which stool donors, including two melanoma patients who received anti-PD-1 antibody therapy and achieved complete remission, were recruited to evaluate the safety and feasibility of FMT combined with anti-PD-1 antibody immunotherapy in 10 patients with refractory metastatic melanoma. Clinical response results were observed in three patients, including two partial responses and one complete response. Moreover, FMT treatment was associated with favorable changes in immune cell infiltration and gene expression profiles in the intestinal lamina propria and TME. In the clinical trial (NCT03341143) conducted by Davar et al., 6 of the 15 patients enrolled in the trial exhibited clinical benefits, including rapid and lasting changes in their gut microbiome and increased abundance of some previously reported gut microbial taxa associated with clinical response, such as *Bifidobacterium. longum*, *Collinsella aerofaciens*, and *E. faecium* [18, 72]. In the TME, increased activation of CD8^+^ T cells and a decreased proportion of myeloid cells expressing IL-8 were observed. In addition, patients treated with FMT had different proteomic and metabolomic characteristics. These two studies also became the first clinical proof-of-concept studies showing that FMT overcomes ICB resistance [172], which greatly expanded the prospects of the clinical application of FMT in antitumor immunotherapy strategies. In addition, there are many other ongoing or completed clinical trials [15, 173–176], which provide strong evidence for the safety and efficacy of FMT regulation of the gut microbiome in the treatment of tumors.

Nevertheless, the compatibility of the donor feces and the recipient intestinal microenvironment during FMT remains to be further ascertained. In addition, there are many nontarget components of donor feces that are transplanted into the recipient’s body, which have unknown effects. Thus, FMT remains a temporary, outcome-oriented solution until the mechanism of action and criteria for use are fully understood. More in-depth mechanistic exploration and the identification of more refined treatment strategies are the main directions of future research.

### Probiotics

The concept of probiotics was first proposed by Metchnikoff [177] and is defined by the Food and Agriculture Organization of the United Nations as “living microorganisms beneficial to the host when injected in sufficient quantities” [178]. However, probiotics that are considered dietary supplements do not need to go through strict review by drug regulatory authorities before being marketed [179], which has resulted in the absence of formulation standards and quality control, exaggerated efficacy and lack of scientific experimental data regarding probiotics [180]. Therefore, while probiotics have been recognized academically for their health benefits, they are still misunderstood by many people. We need to continue to explore the effect and mechanism of probiotics using advanced science and revise the general understanding of probiotic therapy.

Due to the impact of probiotic supplementation on the composition of the gut microbiota, it is rational that the application of probiotics could enhance the host immune response [181, 182], as well as the efficiency of antitumor therapy (Table 3). Lee Se-Hoon et al. found that *B. bifidum* was significantly enriched among the fecal microbes in NSCLC treatment responders in a study of patients treated with different methods, and it was found in an animal model that supplementation with B. bif_K57 combined with oxaliplatin or anti-PD-1 antibody significantly enhanced the antitumor effect. Mechanistic studies showed that the probiotic strain B. bif_K57 could significantly enhance the immune response in the TME and increase the activation of CD4^+^ and CD8^+^ T cells, as well as the secretion of the cytokines IFN-γ and IL-2. These effects may be achieved through the ability of the B. bif_K57 strain to synthesize peptidoglycan [127]. Mager Lukas et al. found that inosine produced by *Bifidobacterium pseudolongum* activates the T-cell-specific A_2A_R signaling pathway and stimulates strong antitumor immunity in the tumor and spleen, thereby promoting the efficacy of anti-CTLA-4 antibodies in mouse colon cancer [134]. In addition to supplementation with *Bifidobacterium*, preclinical studies have found that supplementation with other probiotics, such as *Lactobacillus* [138, 147, 183] or *Streptococcus thermophilus* [139], could significantly improve tumor immunotherapy. In addition, AKK is another species of probiotic that has been discovered and widely accepted in recent years with the development of microbial sequencing technology [184, 185]. AKK, which can induce an adaptive immune response in follicular helper T cells (T_FH_) in Peyer’s patches to maintain intestinal homeostasis, has been reported [78]. Model mice orally supplemented with AKK combined with anti-PD-1 antibody [17] or cisplatin [186] showed apparently improved therapeutic outcomes. Shi et al.’s study revealed that the outer membrane protein of AKK, Amuc, could recruit more tumor-specific cytotoxic T cells in the TME by activating TLR2 signaling and reducing the levels of immunosuppressed Tregs, thus producing a considerable antitumor effect when IL-2 was combined as an adjuvant [131]. These results suggest that beneficial probiotics for health have good prospects for use in research on antitumor effects. In addition to the use of single probiotic strains, it has been suggested that a mixture of multiple strains may be needed to influence the complex microecosystem of the gut. Tanoue et al. isolated 11 strains from the feces of volunteers and mixed them, which could significantly induce the accumulation of IFNγ^+^ CD8^+^ T cells in the intestinal tract of mice, and synergistic ICB produced a significant therapeutic effect in a mouse tumor model; the induction effect was optimal only when all 11 strains were present [187]. These results implied that the interaction of the gut microbiome with the immune system and tumor may involve more complex systemic processes.

**Table 3:** Studies on improving the effect of antitumor immunotherapy by probiotics

Tumor type	Immunotherapy treatment	Species/strain	Overview of mechanism	References
CRC (AOM-DSS/MC38); bladder cancer (MB49); melanoma (B16-F10)	αCTLA-4 mAb	*Bifidobacterium pseudomonas*	Inosine produced by the gut microbiota can translocate to the tumor microenvironment and activated T cells by adenosine A_2A_ receptor combined with costimulation of CpG and IL-12 released by DCs for Th1 differentiation, which results in IFN-γ production and enhanced ICB therapy	[134]
Sarcoma (MCA205); RET melanoma; Lewis lung cancer (LLC)	αPD-1 mAb; αCTLA-4 mAb combined with αPD-1 mAb	*Akkermansia muciniphila*	Oral supplementation with *Akkermansia muciniphila* restored the efficacy of ICB in an il-12-dependent manner by increasing the recruitment of CCR9^+^ CXCR3^+^ CD4^+^ T lymphocytes into tumor beds	[17]
CRC (MC38); Lewis lung cancer (LLC1); breast cancer (4T1)	Oxaliplatin; αPD-1 mAb	*Bifidobacterium bifidum*	Peptidoglycan expressed at high levels in *Bifidobacterim bifidum* can act as TLR2 receptor to stimulate IFN-γ secretion and improve antitumor therapy by αPD-1 mAb or oxaliplatin	[127]
CRC (MC38); melanoma (Braf^V600E^ Pten^−/−^)	αCTLA-4 mAb; αPD-1 mAb	A mixture of 11 strains (11-mix)	An 11-strain mix induces the accumulation of IFN-γ^+^ CD8^+^ T cells through the effects of CD103^+^ DCs in the colonic lamina propria in an MHC Ia dependent manner, thereby activating the antitumor immune response	[187]
Colon cancer	αCTLA-4 mAb	*Lactobacillus acidophilus*	Lysates of *Lactobacillus acidophilus* reduced the number of Treg and M2 cells in tumor-draining lymph nodes and mesenteric lymph nodes, increased the ratio of memory CD8^+^ T cells, and promoted an antitumor immune response	[147]
Melanoma (B16.SIY/B16.F10) bladder cancer (MB49)	αPD-1 mAb	*Bifidobacterium breve* and *Bifidobacterium longum* mixtures	*Bifidobacterium*-derived signals enhanced the effector function of tumor-specific CD8^+^ T cells and promoted an antitumor immune response by modulating DC activation	[190]
Sarcoma (MCA205); RET melanoma; metastatic melanoma; CRC (MC38/CT26)	αCTLA-4 mAb	*Bacteroides fragilis*	The capsular polysaccharides of *Bacteroides thetaiotaomicron* and *Bacteroides fragilis* induce the maturation of lamina propria DCs, combined with the Th1 immune response induced by IL-12 secretion, and promotes the antitumor effect of αCTLA-4 mAb	[71]
Lewis lung cancer (LLC)	Cis-platinum	*Akkermansia muciniphila*	AKK combined with cis-platinum could increase the levels of IFN-γ, IL-6 and TNF-α in peripheral blood and the spleen in mice, inhibit the expression of CD4^+^ CD25^+^ Foxp3^+^ Treg cells, and promote an antitumor immune response	[186]
CRC (CT26)	αPD-1 mAb	*Akkermansia muciniphila*	The glycerophospholipid generation produced by AKK bacteria affects the expression of IFN-γ and IL-2 in tumor microenvironment, resulting in different therapeutic effects of αPD-1 mAb	[148]
Melanoma (B16-F10); CRC (CT26)	Systemic IL-2 therapy	*Akkermansia muciniphila*	Amuc (AKK outer membrane protein) activates antitumor immunity through the TLR2 signaling pathway	[131]
CRC (AOM/DSS, MC38/CT26); Lewis lung cancer (LLC); melanoma (B16-F10)	With or without αPD-1 mAb	*Clostridiales* (*Ruminococcaceae*, *Lachnospiraceae*): *Roseburia intestinalis*; *Eubacterium hallii*; *Faecalibacterium prausnitzii*; *Anaerostipes caccae*	*Clostridiales* promote antitumor immune response in a CD8^+^ T dependent manner	[191]
CRC (MC38)	αPD-1 mAb	*Lactobacillus paracasei* sh2020	Colonization of *Lactobacillus paracasei* sh2020 induces increased CXCL10 expression in tumors, which in turn promotes recruitment of CD8^+^ T cells and promotes an antitumor immune response	[192]
Melanoma (B16-F10); sarcoma (MCA205); CRC(MC38)	αPD-1 mAb; αCTLA-4 mAb	*Enterococcus faecium*; *Enterococcus faecalis*	*Enterococcus* with unique NlpC/P60 peptidoglycan hydrolase activity can produce peptides that activate NOD2 activity and modulate the efficacy of ICB therapy in vivo, promoting antitumor immunity	[193]
CRC (CT26)	αPD-1 mAb	*Lactobacillus rhamnosus*	*Lactobacillus rhamnosus* could effectively restore the gut microbiota depleted by antibiotics, significantly increase the relative abundance of beneficial bacteria, and promote the therapeutic effect of αPD-1 mAb	[194]
CRC (CT26)	Neoantigen cancer vaccine	*Bifidobacterium* (*B. bifidum*, *B. longum*, *B. lactis* and *B. breve*)	*Bifidobacterium* could affect the mechanism of tumor growth, change the composition of the gut microbiota, increase the abundance of antitumor *Muribaculaceae*, reduce the levels of tumor-promoting *Lachnospiraceae*, and promote the antitumor effect of a neoantigen cancer vaccine	[195]
CRC (MC38); sarcoma (MCA205); lung cancer (TC-1)	Cyclophosphamide; αPD-1 mAb	*Enterococcus hirae*	The tape measure protein (TMP) of the probacteriophage found in the genome of *Enterococcus hirae* phage contains an epitope that can bind MHC-I. After treatment with cyclophosphamide or αPD-1 mAb, mice carrying *Enterococcus hirae* developed TMP-specific H-2K^b^ restrictive CD8^+^ T-cell responses that ultimately promoted antitumor immunotherapy	[75]
Melanoma (B16); CRC (CT26)	αTIM-3 mAb	*Enterococcus hirae*; *Lactobacillus johnsonii*	Probiotic administration restored the antitumor activity of αTIM-3 mAb that was impaired by antibiotics usage	[183]
CRC (MC38); lymphoma (EG7)	αCD-47 mAb	Mixture of *Bifidobacterium* species (*B. bifidum*, *B. longum*, *B. brevis*, *B. lactis*)	Systemic administration of *Bifidobacterium* leads to its accumulation in tumors, which can effectively stimulate STING signal transduction, increase the cross-initiation of DCs after αCD-47 mAb treatment, and promote an antitumor immune response	[196]
Breast cancer (4T1); liver cancer (H22)	TGF-β blockade	*Escherichia coli* strain Nissle 1917 (EcN)	EcN colonization could effectively promote tumor-specific effector T-cell infiltration and DC activation after TGF-β blockade, resulting in a stronger antitumor effect	[197]
Melanoma (B16-F10)	αPD-1 mAb	*Lactobacillus kefiranofaciens* ZW18 (ZW18)	ZW18 activated the immunity, promoted tumor CD8^+^ T-cell infiltration, and significantly increased the abundance of *Akkermansia*, the *Prevotellaceae_NK3B31_group* and *Muribaculum*	[198]
CRC (MC38/CT26/HCT116); breast cancer (4T1)	αPD-1 mAb; oxaliplatin	*Lactococcus lactis* GEN3013	*L. lactis* GEN3013 augmented cytotoxic immune cell populations, including CD4^+^ T cells, CD8^+^ effector T cells, and NK cells, in the tumor microenvironment	[199]

The considerable effect of oral probiotics on the antitumor immune response in mice provides a basis for the clinical study of probiotics in the tumor population. In a recent phase I trial (NCT03829111), 29 patients with mRCC were treated for the first time with nivolumab in combination with ipilimumab, and some were supplemented with CBM588, which contains *Clostridium butyricum*. Probiotic CBM588 significantly prolonged PFS in patients with mRCC without additional toxicity and improved response rates to combined ICB therapy [188]. Moreover, the administration of CBM588 to regulate gut microbiota improved the efficacy of ICB treatment in NSCLC cancer patients receiving PPIs [189]. Another analysis of 77 patients with advanced melanoma revealed a positive correlation between the toxicity of anti-PD-1 and anti-CTLA-4 antibodies and the presence of *Bacteroides intestinalis* in the feces of the patients, suggesting that the bacteria could be used as a target for reducing the side effects of ICB treatment [96]. At present, clinical studies on the use of probiotics in the treatment of cancer have been very rare, and the results of only individual clinical trials are not sufficient for cross-validation.

Taken together, these data indicate the potential advantages of probiotics for cancer treatment. However, it is still necessary to improve the quality control of commercial probiotics to confirm their antitumor effect and optimize the strategy for colonization by probiotics. In addition, the specific mechanism of antitumor immunity induced by probiotics needs to be further elucidated.

### Diet and prebiotics

The microbes colonizing the gut can decompose and metabolize physical components that cannot be digested by the host, and the released nutrients can be absorbed by the body [200, 201]. Conversely, diet also affects the gut microbiome and metabolome [202, 203] (Table 4). This interaction has laid a theoretical foundation for the regulation of the bacterial consortium through specific metabolic pathways by dietary formulas.

**Table 4:** Studies on improving the effect of antitumor immunotherapy by prebiotics

Tumor type	Immunotherapy treatment	Prebiotic	Overview of mechanism	References
CRC (CT26); breast cancer (4T1); melanoma (B16-F10)	αCTLA-4 mAb; αPD-1 mAb	Exopolysaccharide (EPS-R1)	Dietary intake of EPS-R1 induces aggregation of CCR6^+^ CD8^+^ T cells in Peyer’s patches and enhances the therapeutic effect of ICBs in tumors in a CCL20-dependent manner	[130]
CRC (MC38)	αPD-1 mAb	Pectin	Pectin changes the composition of the gut microbiota by enriching for butyrate-producing bacteria and promotes butyrate production, which could promote T-cell infiltration and enhance the efficacy of αPD-1 mAb	[132]
Lewis lung cancer (LLC); melanoma (B16F10)	αPD-1 mAb	Ginseng polysaccharide (GPs)	GPs increased the antitumor response to αPD-1 mAb by increasing levels of the microbial metabolites valeric acid and decreasing L-kynurenine levels, as well as the ratio of Kyn/Trp, which contributed to the suppression of Treg cells and induction of T_eff_ cells after combination treatment	[213]
CRC (MC38/CT26); melanoma (B16F10)	αPD-1 mAb	Inulin gel	Oral administration of inulin gel increased the abundance of SCFA-producing microbes and SCFAs, resulting in enhanced memory responses in IFN-γ^+^ CD8^+^ T cells and the formation of stem-like T-cell factor-1^+^ PD-1^+^ CD8^+^ T cells in the tumor microenvironment	[214]
Sarcoma (MCA-205); medullary breast cancer (E0771)	αPD-1 mAb	The berry of *Myrciaria dubia*	Castalagin, an active compound in polyphenol rich *Myrciaria dubia* berries, can enrich for *Ruminococcaceae*, increase the ratio of CD8^+^/Foxp3^+^ CD4^+^ T cells in the TME, and promote the biosynthesis of taurine binding bile acids, which translated into antitumor activity and a stronger anti-PD-1 response	[215]
CRC (MC38)	αPD-L1 mAb	Ultrafine Jujube powder	Ultrafine Jujube Powder consumption resulted in increased abundance of *Clostridium* (including *Ruminococcaceae* and *Lachnospiraceae*), increased SCFA production, and increased infiltration of cytotoxic CD8^+^ T cells in the tumor microenvironment	[191]
Melanoma (B16F10); breast cancer (4T1)	αPD-L1 mAb; Doxycycline	Smectite	Smectite loaded in lactic acid bacteria biofilms inhibits tumor growth and activates DCs through the TLR2 pathway, enhancing the efficacy of chemotherapy or immunotherapy	[217]
RET melanoma; renal cell carcinoma in situ (RENCA-luc); lung cancer in situ (TC-1_luc)	αPD-L1 mAb; αCTLA-4 mAb	Ketogenic diet; 3-hydroxybutyric acid	3-Hydroxybutyric acid inhibited the expression of PD-L1 in ICB-associated myeloid cells, promoted the amplification of CXCR3^+^ T cells, and induced changes in the composition of gut microbiota, such as *Eisenbergiella massiliensis*, thus promoting the antitumor effect of ICB	[218]
Advanced NSCLC	αPD-1 mAb; αPD-L1 mAb	Fluorine-18 fluorodeoxyglucose (^18^F-FDG)	Physiological uptake of ^18^F-FDG by the colon before initiation of ICB was associated with better clinical outcomes and higher gut microbiome diversity in patients with advanced NSCLC	[219]
Solid tumor	αPD-1 mAb	SCFAs	High concentrations of SCFAs in feces were associated with longer PFS	[220]
CRC (MC38)	αPD-L1 mAb	Bilberry Anthocyanin Combo	Bilberry Anthocyanin Combo restored the species diversity of intestinal microbiota, increased the concentration and proportion of butyric acid in feces, enhanced the infiltration of CD8^+^ T cells in tumors, and promoted the effect of αPD-L1 mAb	[221]
CRC (CT26)	αPD-1 mAb	Gegen Qinlian decoction	The combined treatment of Gegen Qinlian decoction and αPD-1 mAb antibody can effectively inhibit the growth of CT26 tumor cells, change the composition of gut microbe, and promote the metabolism of glycerophospholipids and sphingolipids	[222]
Prostate cancer (RP-B6Myc); kidney cancer (RENCA)	αPD-1 mAb	Restricted protein diet	Dietary protein restriction alters the activity of tumor-associated macrophages and enhances their antitumor ability	[223]
melanoma (B16F10)	αPD-1 mAb	Diosgenin	The combination of αPD-1 mAb and diosgenin can induce enhanced response of tumor necrosis and apoptosis	[224]
CRC (MC38)	αPD-1 mAb combined with αCTLA-4 mAb	A standardized extract of cultured Lentinula Edodes Mycelia, AHCC®	AHCC® increased the species abundance of *Ruminococcaceae*, altered the composition of the gut microbiome, and promoted the antitumor effect of anti-PD-1 combined with anti-CTLA-4 immunotherapy by increasing the expression of GZMB and Ki-67 in tumor-infiltrating CD8^+^ T cells	[225]

Geographically, different populations exhibit different dietary habits, which in turn act on the gut microbiota. Accordingly, a prospective trial profiled baseline gut microbiota signatures and dietary patterns among 103 patients from Australia and the Netherlands treated with ICBs for melanoma and performed an integrated analysis with data from 115 patients with melanoma treated with ICBs in the United States [116]. High dietary fiber intake in individuals from areas such as Australia and the United States may lead to greater clinical benefit for those with Bacteroidaceae-dominated microbiomes than in individuals from the Netherlands, who already have fiber-influenced microbiomes. Therefore, diet customization, especially involving dietary fiber, represents a potential strategy for improving tumor therapy. Dietary fiber components are metabolized to produce SCFAs [204, 205], which have been widely reported in the treatment of metabolic diseases [206, 207] and intestinal inflammation [200, 204]. In the last decade, the impact of SCFAs on tumor immunotherapy has also been reported (Table 4). He et al. found that butyrate could promote the therapeutic effect of the immunogenicity drugs oxaliplatin and anti-PD-1 antibody by directly activating antitumor CD8^+^ T cells [133]. However, another study found that a higher level of SCFAs in the peripheral blood may weaken the antitumor immune response induced by anti-CTLA-4 antibodies [208], suggesting that the effect of the metabolites is different in tissues. Therefore, the use of dietary fiber metabolites to regulate antitumor immunity requires more refined research.

Prebiotics are more specific than diet and comprise specific chemicals, which mainly include oligosaccharides and polysaccharides, that promote the growth of specific microbes [209, 210]. Prebiotic usage to promote antitumor immunotherapy has attracted increasing attention in recent years [211, 212]. Huang et al. found that oral administration of ginseng polysaccharide in mice could promote the antitumor effect of ICB, and its internal mechanism was that the supplemented prebiotic could improve the TME and systemic CD8^+^ T-cell function by reshaping gut microbial composition and tryptophan metabolism and inhibiting the effect of Treg cells, leading to the enhancement of the antitumor effect of the anti-PD-1 antibody [213]. In another study using an engineering approach to regulate beneficial microbes in the gut, Han Kai et al. prepared inulin gel that could be orally administered and released site-specifically in the colon to regulate microbial composition in situ, promote SCFA metabolism, induce a systemic T-cell memory response, and enhance the antitumor activity of anti-PD-1 antibody [214]. Furthermore, a recent study showed that a natural polyphenol from the berry of *Myrciaria dubia* could reverse ICB resistance by altering the intestinal microbiome composition [215]. These extracted or modified, one-component natural products can achieve a finer regulation of gut microbes than dietary supplements to a certain extent and offer more possibilities for tumor therapy by targeting the gut microbial structure.

Based on preclinical mouse models, a recently published clinical observational study of 128 melanoma patients treated with ICB found that 37 patients who met the 20 g/day dietary fiber intake threshold had significantly longer PFS than those who did not. Interestingly, the anti-PD-1 antibody showed better therapeutic efficacy in patients with adequate dietary fiber intake and no probiotic supplementation. To investigate the causal relationship between diet and treatment outcome, the authors established a mouse tumor model and found that a lower-fiber diet or supplementation with probiotics consisting mainly of *B. longum* and *Lactobacillus rhamnosus* GG weakened the antitumor effect of the anti-PD-1 antibody [216]. This result, particularly the contrary effect of probiotics, reemphasized the high complexity and specificity of targeting gut microbes to modulate the therapeutic effects of tumor therapy. Fortunately, gut microbes do therefore have potential as targets for antitumor immunotherapy, but the characteristics and mechanisms of action of gut microbes may be more complex than expected.

### Antibiotics and other drugs

The purpose of using antibiotics for tumor patients is to prevent infections by various pathogenic microorganisms, which would inevitably change the composition of the gut microbiota. Therefore, a mass of preclinical studies and clinical cohort retrospective studies have reported that the use of antibiotics weakens the efficacy of tumor immunotherapy, especially ICB therapy (Table 5) [226]. Routy et al. evaluated the effect of antibiotics against PD-1 antibody therapy in 249 patients with advanced NSCLC, RCC, or urothelial carcinoma and found that PFS and OS were significantly shorter in patients who received antibiotics than in those who did not [17]. Chalabi et al. retrospectively analyzed two previous clinical trials investigating patients with metastatic NSCLC (NCT01903993; NCT02008227) and found that both antibiotics and another microbiome altering proton pump inhibitors (PPIs) affected atezolizumab treatment, shortening PFS and OS [227]. These results indicate that the gut microbiome plays an essential role in ICB treatment. Another piece of evidence supporting this idea is that the therapeutic effect of ICB may be affected by the spectrum of antibiotic action. Specifically, patients receiving broad-spectrum antibiotics have a shorter survival than those receiving narrow-spectrum antibiotics [228]. Interestingly, the effect of antibiotics on tumor immunotherapy is also related to the period of patient exposure to antibiotics; antibiotics may have beneficial effects before or 30 days after ICB treatment [229]. These studies showed that antibiotic usage during the ICB treatment period could weaken the tumor treatment effect, but antibiotics are still irreplaceable in preventing infection and postoperative complications after tumor surgical treatment [230, 231]. Hence, the design of an antibiotic treatment plan according to the actual situation of patients, such as an approach determined based on sequencing analysis of the gut microbiome before antibiotic treatment and customized antibiotic formulation, may reduce the disturbances mediated by broad-spectrum antibiotics in the gut microbiota and improve the therapeutic effect.

**Table 5:** Studies on the inhibitory effect of antibiotics on immunotherapy

Tumor type	Immunotherapy treatment	Number of included patients	Antibiotics	References
Melanoma; NSCLC; blood cancer; renal cancer; etc.	αPD-1/PD-L1 mAb, αCTLA-4 mAb, or combination treatment	635	β-Lactam, sulfa, quinolones, macrolides aminoglycosides, tetracycline	[236]
NSCLC; kidney cancer; bladder cancer; head and neck; melanoma	αPD-1/PD-L1 mAb, αCTLA-4 mAb, or combination treatment	102	β-Lactam; fluoroquinolone; cephalosporin; macrolides; sulfonamides	[237]
NSCLC; melanoma; upper airway and digestive tract carcinoma; renal cell carcinoma	αPD-1 mAb	212	β-Lactam; fluoroquinolone; tetracycline class	[238]
NSCLC	αPD-1 mAb; αPD-L1 mAb	531	β-Lactam	[239]
NSCLC; RCC; melanoma; lung cancer; etc.	αPD-1/PD-L1 mAb, αCTLA-4 mAb, or combination treatment	5565	β-Lactam; quinolone; vancomycin; daptomycin; linezoldone; meropenem; azithromycin; furantoin; tetracycline; penicillin; etc.	[240]
NSCLC; melanoma; etc.	αPD-1 mAb; αPD-L1 mAb	196	β-Lactam; quinolone; macrolides; sulfa; tetracycline; aminoglycosides; etc.	[241]
NSCLC; melanoma; RCC; etc.	αPD-1/PD-L1 mAb, αCTLA-4 mAb, or combination treatment	12,492	β-Lactam; fluoroquinolone; penicillins; carbapenems; metronidazole; macrolides; linezolid; vancomycin; ciprofloxacin; meropenem; tetracyclines; levofloxacin	[242]
Metastatic urothelial carcinoma (mUC)	αPD-1 mAb	67	Penicillins; cephalosporins; carbapenems; quinolone	[243]
Muscle-invasive bladder cancer	αPD-1 mAb	149	Fluoroquinolones	[244]
RCC	αPD-1 mAb	93	quinolone; amoxicillin	[245]
Advanced melanoma	αCTLA-4 mAb	1585	Systemic antibiotic	[246]
NSCLC; melanoma; RCC; others	αCTLA-4 mAb; αPD-L1 mAb; αPD-1 mAb	217	Concomitant systemic antibiotics	[247]
Urothelial carcinoma	αPD-L1 mAb	1360	Penicillins; quinolone; cephalosporins; glycopeptide; carbapenems; sulfa; macrolides; aminoglycosides; etc.	[248]
Acute myeloid leukemia (AML); NSCLC; RCC	ICI; chemotherapy	338	Piperacillin; clindamycin; metronidazole. meropenem; vancomycin; furantoin; rifampin. rifaximin; tobramycin	[249]
RCC	αPD-1 mAb	69	β-Lactam; quinolone	[170]
Melanoma	αPD-1 mAb; αCTLA-4 mAb; combined	568	Cephalosporins; penicillins; fluoroquinolone	[250]
Bladder cancer; head and neck carcinoma; melanoma; NSCLC; RCC; sarcoma; others	αPD-1/PD-L1 mAb, αCTLA-4 mAb, or combination treatment	690	β-Lactam	[251]
NSCLC; melanoma; urothelial carcinoma; RCC; lung cancer; sarcoma	αPD-1/PD-L1 mAb, αCTLA-4 mAb, or combination treatment	2740	Antibiotics	[252]
NSCLC	αPD-1 mAb;	234	β-Lactam; cephalosporins; quinolone	[253]
Melanoma	αPD-1 mAb; αCTLA-4 mAb; combined with chemotherapy	74	β-Lactam	[254]
NSCLC	αPD-1 mAb; αCTLA-4 mAb	109	β-Lactam; fluoroquinolone; etc.	[255]
NSCLC; RCC	Anti-PD-L1	360	β-Lactam; quinolone; sulfa	[81]
mRCC	αPD-1/PD-L1 mAb; mTOR inhibitors; IFN-α; VEGF-TT	4290	β-Lactam; fluoroquinolone; macrolides; tetracyclines; etc.	[256]

Nevertheless, there does not always seem to be a positive correlation between bacterial burden and treatment outcomes. In a retrospective review of 57 patients with MSI-H/dMMR mCRC receiving anti-PD-1 mAb, there was no association of lower response rates or survival in those patients exposed to antibiotics [232]. Gut microbes can relocate into surrounding tissues and influence tumor progression. Broad-spectrum antibiotic depletion of the gut microbiome prevented invasive PDAC and enhanced the antitumor immune response [32]. These results showed that the role of the gut microbiome in tumor therapy was contrary to previous results, suggesting that an excessive intestinal microbe load or a high concentration of microorganisms in tumors may have adverse effects on tumor therapy. Etiologically, the studies that observed negative effects of the microbiome on therapy outcomes have involved studies of pancreatic cancer [233, 234], and one possible explanation is that the pancreas and gut are anatomically connected through the pancreatic duct [235]. The continuity of the two may lead to a higher microbial load in the pancreas than in other tissues, which is related to the occurrence and immunosuppression of pancreatic cancer. These results suggest that the different benefits of the gut microbiome on different tumor types may be related to the distribution of the microbiome in different organs.

## Future prospects: opportunity and challenge coexist

With the gradual increase in attention towards the important role of the gut microbiome in human health, research on the formation, development, diagnosis and treatment of tumors is steadily advancing to a new stage. Due to the widespread application of NGS, metabolomics, proteomics and other multiomics techniques, researchers have gradually gained a clearer understanding of the colonization, transfer and recolonization of microbes in various tissues and organs of the body and identified the close relationship of function and causation between health and disease states and the colonization of microbes in tissues, including those in the TME. Finally, the concept of the “polymorphic microbiome” has been regarded as a new hallmark of cancer [21].

Nevertheless, some substantial problems exist that prevent gut microbes from acting as more potent weapons against a variety of diseases, including tumors. First, due to the limitation of sample allocation, the collected sample information cannot truly reflect the microbial composition of the gut in space [257]. It is still to be discussed that stool samples represent the bacterial colonization in the niche of the gut-intestinal tract, and it is even more difficult to extract microbiota from tumors or other focal sites. In addition, there are also many obstacles to monitoring the temporal changes in the gut microbiome during disease progression or treatment because the gut microbiota is easily affected by diet, environment, host age, gender, lifestyle and other factors, which reflect the collective changes in multiple factors. Therefore, it is difficult to determine the causal relationship between the gut microbiome and diseases. The second is the functional diversity of the gut microbiota. For example, taxonomically, bacteria of the same species differ greatly in function. In addition, the same bacteria may show different characteristics due to environmental changes or the composition of other bacteria, and different bacteria may share similar metabolic pathways with similar functions [47, 193]. Hence, the interactions between bacteria should not be ignored. Finally, there is a diversity of data and differences in analysis techniques. Although there is a wealth of multiomics data, including human samples, indicating the microbiome associated with disease and treatment outcomes, reproducibility of results across research projects has been difficult to achieve (Table 1) [258]. This may be due to, on the one hand, the differences that researchers collect or preserve samples for research objects; on the other hand, the generation of many multiomics data makes the computational inference of data a challenge, accompanied by redundant data analysis methods. The solution to this problem may require standard analytical manual in the future, including machine learning and artificial intelligence, as well as better data and resource-availability mechanisms.

## Conclusion

The existing research results have provided substantial evidence for the conclusion that the commensal microbiome impacts the efficiency of antitumor immunotherapy, and promoting the effect of antitumor immunotherapy by modulating the composition of gut microbiota has been shown to work. However, due to the variation in individual microbiomes and limitations of complex multiomics analysis, there is a lack of systematic research on the factors of the microbiome that are involved in antitumor therapy, and mutual authentication among study conclusions cannot be obtained. Moreover, precise mechanistic research on the impact of the microbiome on antitumor immunity is still scarce. As a result, the manipulation of the commensal microbiota by modern medical techniques for the treatment of diseases, including tumors, is still far from clinical application.

Nonetheless, it is optimistic that considerable resources are being devoted to research that links commensal microbiota and host health and disease. Preclinical and clinical studies have demonstrated that regulation of the gut microbiota may improve the efficacy of antitumor therapy from multiple perspectives and at multiple levels. In addition to knowledge regarding the individual differences in the commensal microbiome and the diversity of influencing factors, individual multiomics analysis combined with precision medicine, instead of broad FMT or probiotics, will inevitably become the future direction of the development of tumor immunotherapy or treatment for other diseases affected by the microbiome.

## Data Availability

Not applicable.

## Abbreviations

PFS: Progression-free survival
OS: Overall survival
NGS: Next-generation sequencing
ICB: Immune checkpoint blockade
ACT: Adoptive T-cell therapy
GF: Germ-free
PRRs: Pattern recognition receptors
TLRs: Toll-like receptors
MAMPs: Microbe-associated molecular patterns
LPS: Lipopolysaccharides
DCs: Dendritic cells
TJ: Tight junction
TME: Tumor microenvironment
SCFAs: Short-chain fatty acids
Møs: Macrophages
NK cell: Natural killer cell
GPCR: G-protein-coupled receptor
HDACs: Histone deacetylases
IFN-γ: Interferon-γ
GZMB: Granzyme B
BA: Bile acid
TCRs: T-cell receptors
CTLA-4: Cytotoxic T-lymphocyte-associated antigen 4
PD-1: Programmed cell death protein 1
PD-L1: Programmed cell death protein 1 ligand
irAEs: Immune-related adverse reactions
FMT: Fecal microbiota transplantation
PAMPs: Pathogen-associated molecular patterns
NSCLC: Non-small cell lung cancer
TMAO: Trimethylamine *N*-oxide
AhR: Aryl hydrocarbon receptor
A_2A_R: Adenosine 2A receptor
TILs: Tumor-infiltrating lymphocytes
CTLs: Cytotoxic T lymphocytes
CARs: Chimeric antigen receptors
MHC: Major histocompatibility complex
AKK: *Akkermansia muciniphila*
PPIs: Proton pump inhibitors
PDAC: Pancreatic ductal adenocarcinoma
BDSCs: Myeloid-derived suppressor cells
CLRs: C-type lectin receptors
